# Coffee Grounds-Derived CNPs for Efficient Cr(VI) Water Remediation

**DOI:** 10.3390/nano11051064

**Published:** 2021-04-21

**Authors:** Simona Bettini, Michela Ottolini, Rosanna Pagano, Sudipto Pal, Antonio Licciulli, Ludovico Valli, Gabriele Giancane

**Affiliations:** 1 Department of Biological and Environmental Sciences and Technologies, DISTEBA, University of Salento, Via per Arnesano, I-73100 Lecce, Italy; rosanna.pagano@unisalento.it (R.P.); ludovico.valli@unisalento.it (L.V.); 2 Consorzio Interuniversitario Nazionale per la Scienza e Tecnologia dei Materiali, INSTM, Via G. Giusti, 9, I-50121 Firenze, Italy; 3 Department of Engineering for Innovation, University of Salento, Via per Monteroni, I-73100 Lecce, Italy; michela.ottolini@unisalento.it (M.O.); sudipto.pal@unisalento.it (S.P.); antonio.licciulli@unisalento.it (A.L.); 4 Department of Cultural Heritage, University of Salento, Via D. Birago, 48, I-73100 Lecce, Italy

**Keywords:** carbon dots, carbon nanoparticles, Cr(VI) remediation, hexavalent chromium, spent coffee grounds

## Abstract

Carbon nanomaterials are a group of materials characterized by sp^2^/sp^3^ carbon backbone which, combined with surface atoms and/or chemical groups, ensures peculiar physical chemical features for a wide range of applications. Among these materials, carbon dots and carbon nanoparticles belong to carbon nanomaterials with a few nanometer dimensions. In this work, carbon nanoparticles were produced from spent coffee grounds as sustainable carbon source through a simple, cheap and eco-friendly procedure according to an oxidation process (at controlled temperature) driven by hydrogen peroxide. Atomic Force Microscope (AFM) and fluorescence, UV-Vis absorption, FT-IR and Raman spectroscopy were used to assess the formation of carbon nanomaterials of about 10 nm with the typical emission and absorption properties of carbon dots and peculiar surface features. In fact, the presence of heteroatoms, i.e., phosphorus, and the carbonyl/carboxyl surface groups on carbon nanoparticles, was proposed to confer peculiar properties allowing the fast Mn(VII) reduction to Mn(II) at neutral pH and the Cr(VI) reduction to Cr(III) in weak acid aqueous media.

## 1. Introduction

The increasing and unusual episodes concerning emerging pollutant effects on water purity and availability have prompted a remarkable enhancement in investigations aiming at facing and limiting these novel challenges. The remediation to this contemporary society problem has involved several approaches such as adsorption onto disparate kinds of adsorbents [1], filtration [2], flotation [3], ion exchange [4], microbiology methods [5], photo-induced redox reactions [6], precipitation [7], reverse osmosis [8], photocatalysis [9] and so on.

Among contaminants, harmful heavy metals tend to accumulate and show pronounced permeation properties. They are usually derived from anthropogenic activities, mainly in industrial and agricultural areas. Taking into account the extensive applications of transition metals in manufacturing processes, their consequent pollution problems demand pressing and priority solutions. In this research, we have undertaken investigations with the goal of demonstrating the potentialities of our green-synthesized carbon dots in the remediation of criticisms induced by transition metals such as Chromium and Manganese in aqueous environments.

In the large family of carbon dots (CDs), it is possible to gather together all the materials mainly composed of carbon atoms and with dimensions of a few nanometers [10]. From a chemical point of view, CDs consist of sp^2^/sp^3^ carbons and oxygen and/or nitrogen containing pendant groups. Several species belong to the CDs family: graphene quantum dots [11], carbon nanotubes [12,13], fullerenes [14], polymer dots [15] and carbon nanodots (CNDs) [16,17]. In particular, CNDs can be further divided into carbon quantum dots (CQDs) and carbon nanoparticles (CNPs) [18]. Both CQDs and CNPs are spherically shaped though CQDs have a crystal lattice; in contrast, CNPs do not show any crystal lattice [19].

CNDs show very interesting photoluminescence features [19] that propose such materials for several applications. For example, they can be used as a bioimaging and theranostic agent [20], as a hole transporter layer [21] or as a photoactive material in solar cell devices [22]; they can also promote the formation of singlet oxygen for anticancer applications if opportunely doped [23]. Also, they can be used as photocatalysts [24,25,26], in sensing and biosensing applications [27,28] and in environmental pollution control [29]. CDs can be obtained by means of a top-down approach or bottom-up method [30,31]. In the first case, carbon atoms are cut from graphene oxide, nanotubes or carbon fibers. In the bottom-up method, carbon-based materials [32], such as hexabenzocoronene [33] arginine [34] and urea [35] are used as the carbon source. 

In the present manuscript, the bottom-up approach has been used to obtain CNPs starting from coffee grounds as a carbon source according to a relatively simple and rapid pyrolysis-based procedure. The obtained nanostructures were used to efficiently convert the well-known genotoxic cancerogenic agent Cr(VI) ion into the more environmentally friendly Cr(III). Hexavalent chromium can be absorbed by the lung and gastrointestinal tract [36] and when internalized in the cells, it can react producing reactive intermediates capable to attack DNA, proteins and lipidic membranes, thereby affecting the cellular vitality and integrity. It is worth underlining and stressing that water remediation from toxic ions and compounds has developed in recent years as a hot and particularly emotive and controversial topic [37,38,39,40]. Most of the remediation methods regarding Cr(VI) reported in the literature are based on reduction to Cr(III) by oxidation-reduction reactions in the presence of elemental Fe, Mn(II), S^2−^, organic reducing compounds and a biological approach using bacterial strain [41,42,43]. Here, CNPs obtained from waste material have been used to quickly and effectively convert Cr(VI) into Cr(III), proposing the present approach in the frame of a circular economy and sustainable treatment and remediation of water sources. The environmentally and biologically compatible proposed carbon nanomaterials are suggested here as a proof-of-concept for the treatment of industrial and potable water for the remediation from Cr(VI) pollution.

## 2. Materials and Methods

### 2.1. Materials

Spent coffee grounds were used as the carbon precursor. Acetic acid (glacial, ACS reagent, ≥99.7%,), sodium chromate tetrahydrate (99%), potassium permanganate (ACS reagent, ≥99.0%) and manganese (II) sulfate monohydrate (ACS reagent, ≥98%) were purchased from Sigma-Aldrich (St. Louis, Missouri, US) and used as received without any further purification. All the experiments were carried out using ultrapure MilliQ grade water.

### 2.2. Synthesis Methods of CNPs

The approach adopted in this work consists of two steps: first, coffee ground powders were carbonized at 600 °C for one hour; in the second step, 0.1 g of carbonized coffee grounds were dispersed in 10 mL of ultrapure MilliQ grade water and mixed with 150 µL of H_2_O_2_ (30%) under sonication for 20 min, allowing oxidation events to start. Then, the reaction mixture was heated at 150 °C for 90 min. Then, it was left to cool at room temperature and the volume was again adjusted to 10 mL by adding ultrapure MilliQ grade water. The dark brown suspension obtained was centrifuged (8000 rpm for 20 min) and filtered (0.45 μm hydrophilic filter), yielding a yellowish colloidal solution typical of CDs with a concentration of about 0.5 g L^−1^.

### 2.3. CNPs Characterization

Fluorescence spectra were acquired by a fluorescence spectrophotometer (Horiba Jobin Yvon Fluorolog) by changing excitation wavelength in the range 300–420 nm for the down conversion experiments and between 700 and 800 nm for the up-conversion measurements. UV-Visible measurements were carried out by using a Perkin Elmer Lambda 650 spectrophotometer. The Fourier Transform Infrared (FT-IR) spectroscopic characterization was performed by using a PerkinElmer Spectrum One IR spectrometer in Attenuated Total Reflectance (ATR) mode [44]. Each spectrum is the average of 32 scans (4 cm^−1^ resolution) in the range from 800 to 4000 cm^−1^. Lapped Si/SiO_2_ substrate was used to deposit by drop casting the samples for the morphological analysis by means of an Atomic Force Morphology (AFM) instrument (SmartSPM 1000 AIST-NT HORIBA). Micro Raman Xplora (Horiba) with a laser at 532 nm was used for acquiring Raman spectra.

X-ray Diffraction (XRD) measurements were carried out on the as synthesized sample using Rigaku Ultima diffractometer (Cu Kα radiation, λ = 1.5406 Å) operating at 40 kV/20 mA with the step size of 0.02°. For this purpose, a multilayer film was deposited on the polished silicon wafer by the drop casting method from the aqueous suspension of CNPs, followed by drying. Elemental analysis on spent coffee grounds and CNPs was carried out by the X-ray Fluorescence spectroscopy (XRF) technique by using a M4 TORNADO Micro-XRF spectrometer (Bruker Nano, Berlin, Germany) operating at 50 kV/600 µA (30 W) equipped with an X-Flash solid state silicon drift detector. The grounded coffee powders were directly measured by making a pellet when CNPs were drop casted on Silicon wafer to form a multilayer film.

ζ-Potential measurements of as synthetized CNPs and after the remediation experiments were performed using a Malvern Panalytical Nano ZS Zetasizer instrument (Malvern Panalytical Limited, Malvern, UK).

## 3. Results

**Figure 1:**
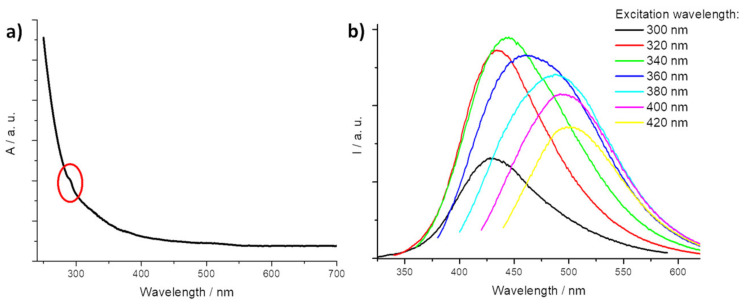
(**a**) UV-Visible spectrum of CNPs. In the red oval, the transition band n–π* is highlighted. (**b**) Fluorescence emission obtained irradiating the CNPs solution with different excitation wavelengths.

The UV-Visible spectrum of CNPs dissolved in ultrapure water solution was recorded and it is reported in Figure 1a. The typical spectral profile of CDs exhibits a weak absorption band located at 290 nm, imputable at n–π* transition of C=O and N=O groups [45,46].

Emission spectra, obtained by excitation at different wavelengths, were recorded (Figure 1b). The characteristic emission features of CNPs were observed when the excitation wavelength was changed from 300 nm to 420 nm with a step of 20 nm. The maximum intensity of photoluminescence emission for 0.05 g L^−1^ CNPs aqueous solution was located at about 445 nm upon 340 nm excitation with a monotonic intensity increasing from 320 nm to a 340 nm excitation wavelength [47]. The photoluminescence phenomenon of CDs is still debated and is usually attributed to both the surface states (depending on hybridization of the carbon backbone and chemical groups on the dots surface) and to the conjugated π-domains, which are determined by the carbon core [19]. The synthesized CNPs exhibited good up-conversion fluorescent properties with the emission peak position depending on the excitation wavelength, with a maximum located at about 500 nm for an excitation wavelength at 800 nm (Figure S1). 

**Figure 2:**
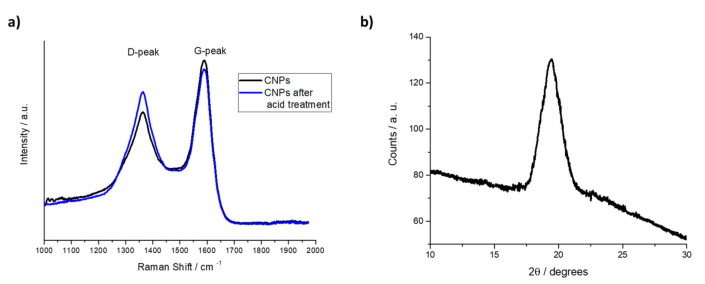
(**a**) Raman spectra of as-synthetized CNPs and after 1 h acid treatment at pH 4.5 (**b**) XRD spectrum of as synthetized CNPs.

Raman spectrum obtained using a 532 nm excitation laser is reported in Figure 2a and shows the typical presence of D and G bands located at 1360 and 1590 cm^−1^, respectively. The D-peak in the Raman spectrum is usually attributed to the contribution from defects, surface functionalization groups and edge effect. On the other side, the G-band corresponds to the graphitization associated with the CNPs [48]. The intensity ratio between the G-peak and D-peak is about 1.2 and it suggests that the as-synthetized CNPs are partially composed of a crystalline graphitic structure. The formation of carbon dots was confirmed by X-ray diffraction performed at low θ (Figure 2b) that clearly shows a broad diffraction peak at about 20° imputable to the amorphous carbonic structure [49]. No sharp peak was observed in the XRD patterns, confirming the amorphous character of the synthetized carbon nanostructures [50].

The CNPs composition was investigated by means of X-ray fluorescence and FT-IR spectroscopy. XRF highlights the presence of phosphorous both in the starting material (the coffee grounds) and in the CNPs. After the synthesis procedure, the presence of contaminant elements such as calcium, sulfur and iron is strongly reduced (Table 1); in contrast, P and Mg atoms are still present in the formed CNPs (Table 1). 

**Figure 3:**
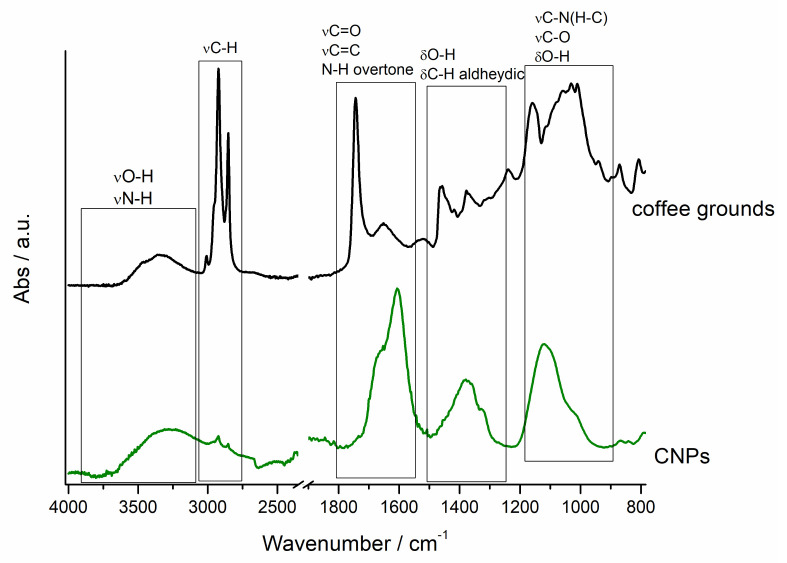
FT-IR spectra of coffee grounds and CNPs.

FT-IR spectroscopy was performed to characterize the overall synthesis procedure starting from the raw material, coffee grounds, up to the final product, CNPs. The black line in Figure 3 corresponds to IR features of coffee grounds and is in perfect agreement with the literature [36]. The spectrum is characterized by the presence of the typical lignocellulosic materials vibrations in the region comprised between 3600 and 3200 cm^−1^ due to the O-H stretching mode and between 1100 and 950 cm^−1^ due to C-O stretching modes of cellulose, hemicellulose and lignin [51,52]. The C-H stretching mode frequency range (3015–2850 cm^−1^) evidences several and intense contributions due to CH vibrations of alkyl chains of fats and of xanthine derivatives (i.e., caffein and chlorogenic acid) [53,54]. Further, diverse signals arising from caffeine or in general xanthine content are evident and ascribable to C=O, C-N and N-H groups: at 3130 cm^−1^ the N-H stretching; at 1745 cm^−1^ the imidazole C=O; at 1660 cm^−1^ the carboxyl asymmetric stretching mode; at 1260 and 1155 cm^−1^ C-N stretching of xanthine side chain and imidazole, respectively; between 950 and 800 cm^−1^ N-C-H deformations of xanthine side chains [55]. 

The carried-out reaction entails an oxidation process allowing precursor fragmentation and the next nucleation and growth of CNPs, and accordingly remarkable changes of FT-IR features of the formed CNPs (green line Figure 3). In fact, at between 3630 and 3020 cm^−1^ a broad band arising from OH and NH stretching modes is exhibited, between 3000 and 2800 cm^−1^ C-H stretching arising from both graphitic and aliphatic carbon is evident, between 1670 and 1590 cm^−1^ range contributions that can be assigned to C=O (partially carboxylic and partially aldehydic) stretching vibration and a combination of C=C stretching and NH overtones are apparent [56], between 1380 and 1310 cm^−1^ O-H bending and aldehydic C-H bending are observable and finally in the range 1150–950 cm^−1^ C-NH-C stretching [57] and carboxylic moieties C-O stretching modes are detectable.

**Figure 4:**
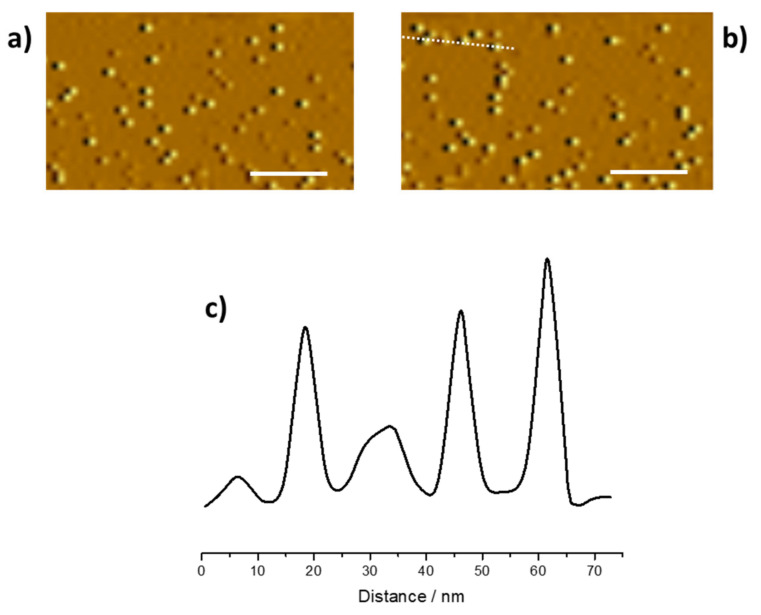
AFM images of the CNPs (**a**) as synthetized and (**b**) after acid treatment (pH 4.5 for 1 h). The bar in the figures is 50 nm wide. Image (**c**) reports the carbon dots z-profiles related to image CNPs in image (**b**) crossed by the white line.

The morphology of the synthetized CNPs was studied by means of atomic force microscopy and it was compared with the carbon nanoparticles images after acidification (Figure 4a,b). The AFM image of the as-synthetized CNPs shows spherical structures of about 10 nm (see the inset image in the Figure 4c) according to the literature data about carbon dots that were obtained with similar synthetic procedures [58]. The morphology of the nanostructures did not appear to be influenced by acid treatment (Figure 4b), as they still showed spherical structures of about 10 nm. In contrast, a ζ-potential investigation performed on the as-synthetized CNPs showed a surface charge of about −12 mV that increases up to −24.7 mV after the treatment of the carbon dots with acetic acid for 1 h at pH 4.5. The interaction with chromium ions, Cr(VI) did not influence the surface charge or the exposure to permanganate (Figure S2).

The effect of the acid treatment on the Raman signals is evident when comparing the G/D band ratio of the bare CNPs and the acid treatment performed on the carbon nanostructures (Figure 2). The intensity ratio between G-peak and D-peak changed from 1.2 in the case of the as-synthetized CNPs to 1.07 after 1 h acid treatment, suggesting that the number of defect sites on the surface of CNPs increases under the acid attack. On the other side, the intensity ratio between G-peak and D-peak is not influenced by the presence of Cr(VI) in the solution (Figure S3a). It is very interesting to observe that the effect of permanganate ion is very similar, and even stronger, in comparison with that one observed in the case of the acid treatment. When the carbon dots are exposed to potassium permanganate, the G-peak/D-peak intensity ratio decreases down to 1.04 (Figure S3b).

FT-IR investigations on CNPs treated at pH 4.5 for 1 h, CNPs treated with Cr(VI) at both pH 6.6 and pH 4.5 and of CNPs treated with Mn(VII) at pH 6.6 are all reported in Figure S4. The experimental results suggest that the signals arising from C=O (with a shift towards higher wavenumbers at about 1700 cm^−1^), C-O, O-H and C-H aldehydic vibrations are influenced at pH 4.5, suggesting the formation of a larger amount of carboxylic moieties: two peaks at 1418 cm^−1^ and 942 cm^−1^ arising from in plane and out of plane modes, respectively, of carboxylic OH are evident (red line Figure S4). This behavior is still visible in the presence of Cr(VI) at pH 4.5, even if these two signals become substantially less pronounced. The FT-IR features of CNPs, instead, in the presence of the two oxidizing ions at pH 6.6, remain almost unchanged. These observations are in very good agreement with the obtained results from ζ-potential. Fluorescence features of CNPs are strongly influenced by the solution pH. The photoluminescence of CNPs at natural pH (pH = 6.6) appears quenched by the presence of sodium chromate (10^−4^ M). It is interesting to observe that when the sodium chromate is dissolved in CNPs water solution at pH 4.5, the fluorescence intensity is reduced of the same amount observed for the CNPs dissolved in water at pH 4.5 (Figure S5). In fact, the fluorescence emission of CNPs is slightly influenced by the presence of Cr(III) ions in solution.

The effect of potassium permanganate perfectly fits the previously observed spectroscopic behaviors. In fact, Mn(VII) induces a remarkable quench of CNPs emission (10^−4^M); in contrast, Mn(II), incapable of modifying the CNPs surface, induces only an almost negligible quench of CNP fluorescence (Figure S6).

**Figure 5:**
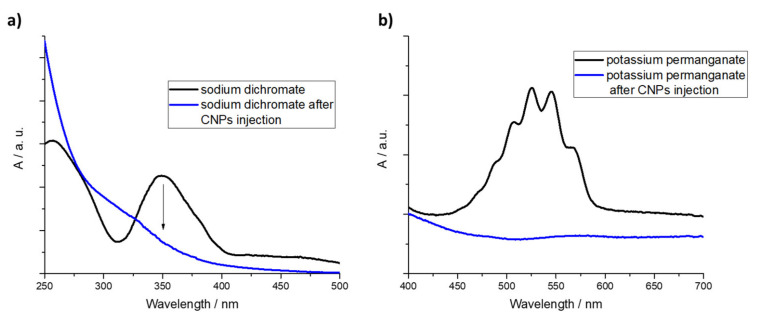
Spectroscopic evidence of the effect induced by CNPs on (**a**) dichromate and (**b**) permanganate solutions.

More interesting than the spectroscopic changes induced by dichromate and permanganate ions on CNPs are the effects of CNPs on the dissolved Cr(VI) and Mn(VII). In fact, the presence of dichromate was monitored by means of UV-Visible spectroscopy considering the typical absorption band located at 350 nm (Figure 5a). When even a small amount of dots (0.05 mg mL^−1^) is added to the aqueous solution containing Cr(VI), the typically yellowish color of the solution immediately changes, for few seconds, in a green colored solution and then turns violet. As will be more deeply discussed in the next section, this suggests that the chromium ions change their oxidation state from +6 to +3. Analogous behavior has been observed in the case of permanganate (Figure 5b).

## 4. Discussion

CNPs have been prepared by using as starting materials spent coffee grounds as carbon source in the frame of circular economy and sustainable chemistry; furthermore, coffee grounds are reported to have potential toxic effects on human health and environment [53]. The developed synthetic approach requires two steps: first, the waste has to be powdered and carbonized; then, a H_2_O_2_ assisted pyrolysis is carried out in order to induce the strong oxidation processes allowing fragmentation, polymerization and nucleation of CNPs, as expected from similar reported procedures [59]. Moreover, the relatively mild conditions (150 °C and ambient pressure) permit us to preserve the presence of some natural doping agents that can influence the physical-chemical features of the formed CNPs [60].

Doped and undoped CNPs are usually used for their peculiar spectroscopic features. On the other side, the spectroscopic characteristics of a chemical compound are, in the widest sense, evidence of the electronical properties of the materials. The electronic cloud of the CNPs, similarly to other allotropic forms of carbon, such as fullerenes and carbon nanotubes, gives these compounds high chemical reactivity towards several species and proposes the CNPs for a plethora of applications [61]. In the present contribution, the synthetized carbon dots were successfully used for the treatment of pollutants dissolved in water.

### 4.1. CNPs Spectroscopic Characterization

The defect sites on CNPs surface act as active sites for promoting the oxidation process. [62]. Loss of crystalline structure on the carbon dots surface has been evidenced by the vibrational spectroscopy.

The surface chemical composition is reported to be fundamental for the further applications of CNPs; FT-IR is a powerful approach for achieving this kind of information. So, dehydrated coffee grounds and formed CNPs were both characterized by this technique. The results further indicate the complexity of the starting material, which is a mixture of different organic compounds, such as cellulose, lignin, hemicellulose, xanthine and fats (in a different percentage) [53], with an ash and mineral content as highlighted by XRF analysis.

Important information about the CNPs structure, size and dimension can be obtained from the CNPs Raman spectra. The position of the D peak is not related to the CNPs dimension, instead the G band position and the full-width half maximum (FWHM) of the G band are connected to the dot size [63]. In particular, for CNPs below 17 nm, the G band shifts toward higher frequencies as the CNPs dimension increases and FWHM value decreases [64]. In the present case, the G band position suggests that spherical nanostructures with dimensions comprised of 8 nm and 10 nm are formed and it is confirmed by the FWHM that results for the G band are equal to 116 cm^−1^ [63].

The infrared vibrational approach allows understanding that the CNP surfaces, upon the carbonization and oxidation step, are essentially characterized by the vibrations of these chemical groups: C=C, C=O, OH, NH. In particular, aldehydic and alcoholic groups can be hypothesized to decorate CNP surfaces and contribute to a partial negative charge, as validated by ζ-potential results that will be discussed hereafter.

The effect of acid treatment on the CNPs aims to induce the formation of further defect sites on the carbon surface, as confirmed by both FT-IR and Raman techniques. In particular, the D-band increases in comparison with the G-band when the dots are placed in acid environment. The acid attack reduces the order of the nanostructures, generating new defects and new active sites.

The formation of new defects on the dots surface was also confirmed by monitoring the ratio of G band intensity (I_G_) to the D band intensity (I_D_). The I_G_/I_D_ decreases from 1.2 in the Raman spectrum of the as-synthetized CNPs to 1.07 after 1 h acid treatment at pH 4.5 (reached adding acetic acid drop-by-drop). The disorder D-band is related to defects in the graphite lattice (i.e., sp^3^ carbon atoms), and the G-band is related to sp^2^-hybridized carbon atom networks [65,66]. Furthermore, peak position and FWHM of G band are not influenced by the acid attack, suggesting (this is further confirmed by morphological characterization, Figure 4b) that the procedure does not influence the CNPs size but it is focused on the CNP surfaces.

ζ-potential measurements furthermore support the surface modification induced by the acid attack. The surface charge variations from −12 mV to −24.7 mV confirm the rationale proposed by FT-IR analysis that underlines the presence of more carboxylic moieties on the surface after the acid treatment.

In contrast, the morphology of the CNPs seems to not be influenced by the acid action and the shape and dimension are preserved.

### 4.2. Remediation Experiments

For the treatment of chromium ions, different pH values have been used. In the first experiment, the sodium chromate (10^−4^ M) was completely converted in sodium dichromate at pH 2.5 (Figure 5a). When the CNPs were added to the aqueous solution (100 µL 0.05 mg mL^−1^ concentration), a rapid bleaching of the absorption band of Na_2_Cr_2_O_7_ at 350 nm is recorded. The yellow-orange colored solution quickly bleaches and a colorless solution is generated. 

**Figure 6:**
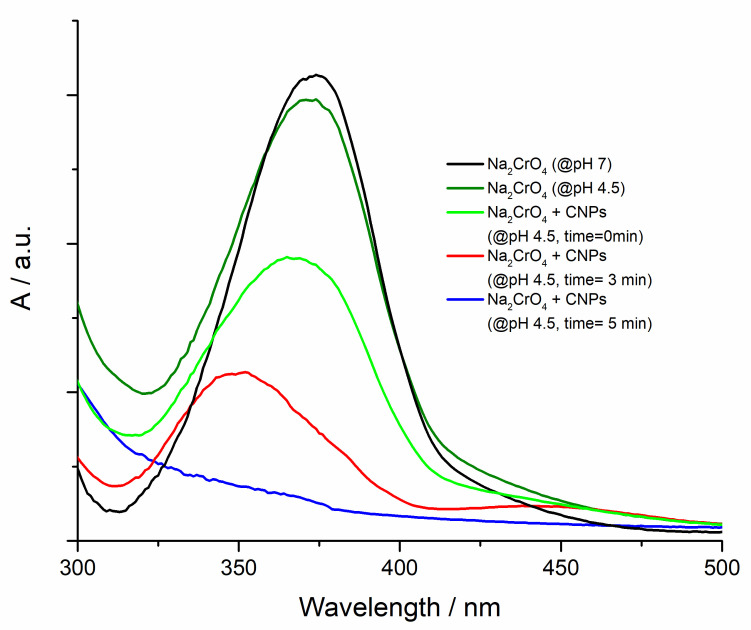
CNPs effect on Vis spectrum of Cr(VI) acid solution during the treatment and Vis spectrum of Cr(VI) at neutral pH in presence of CNPs (black line) for comparison.

A very interesting result is obtained starting from a chromate/dichromate (10^−4^ M) solution at pH 4.5. In this condition, an equilibrium between chromate and dichromate is obtained. When the CNPs were added to the solution (100 µL 0.05 mg mL^-1^ concentration), the absorption band of chromate centered at 350 nm simultaneously decreases its intensity and shifts towards lower wavelengths (Figure 6). It is worth highlighting that an isosbestic point cannot be observed, thus testifying the participation of more than two species to the equilibrium. This can be explained as follows: at the initial stage, before the CNPs injection, a slightly acidic environment (pH 4.5) promotes both the formation of new active sites on the CNPs carbon dots and the establishment of a new chromate/dichromate equilibrium. Presumably, the acid environment converts aldehydic into carboxylic moieties, as suggested by the FT-IR analysis (Figure S4), and this promotes the ion reduction. The sodium dichromate interacts with the carbon dots oxidation sites, reducing the chromium oxidation state from +6 to +3. The FT-IR features of CNPs at pH 4.5 upon the reduction of Cr(VI) indeed suggest the involvement of the carboxylic groups in this process, since the signals at 1418 cm^−1^ and 942 cm^−1^ arising from carboxylic OH appear to be quenched. This step reduces the dichromate concentration and, therefore the band at 350 nm is reduced. So, at least three species simultaneously exist in the solution and they are not in chemical equilibrium, as demonstrated by the blue spectrum in Figure 6 where both chromate and dichromate ions are reduced to Cr(III) by the mediation of CNPs. Fluorescence emission of CNPs at different pH values in the presence of sodium chromate confirms the proposed rationale (Figure S5). In fact, the acid environment strongly influences the CNPs fluorescence emission as a consequence of the creation of new defect sites on the carbon dots. When the sodium chromate is added to CNPs solution at pH 4.5, no further quenching is observed since, as suggested by visible spectroscopy, the Cr(VI) is converted in Cr(III). Chromium(III), at the same concentration of the sodium chromate used to monitor the fluorescence alteration of the CNPs, only slightly influences their photoluminescence (Figure S5).

The chemical reduction of permanganate can be achieved without any acid mediation. In fact, it is reported that KMnO_4_ promotes new defect sites on dot sites [62] and, according to the proposed rationale, the reduction of permanganate ion in the colorless Mn^2+^. This behavior does not induce chemical changes on CNPs surface, according to FT-IR and ζ-potential investigations. The Mn oxidation state change was observed by means of UV-Visible spectroscopy (Figure 5b) by monitoring the rapid bleaching of the typical band of permanganate ion. As was reported in the case of Cr(VI), fluorescence measurements (Figure S6) confirms the rationale pointed out by means of UV-Visible spectroscopy. Mn(VII) is able to promote the formation of new defect sites on the CNPs, activating the carbon allotropes for the permanganate reduction, and this phenomenon (analogously to the acid effect) induces a photoluminescence quenching. Mn(II) is not able to modify the CNPs, so the carbon dots fluorescence is weakly influenced by the presence of Mn(II) ions.

## 5. Conclusions

Spent coffee grounds have been successfully used as a carbon source for synthetizing CNPs by an eco-friendly approach working at 200 °C and at ambient pressure for 90 min in MilliQ grade water. The complex chemical and mineral composition of coffee grounds, ensuring their peculiar optical properties, have been deeply investigated and exploited for the fast reduction of Cr(VI) to Cr(III) in aqueous media in a weak acid environment. The Cr(VI) remediation process based on the synthetized CNPs has been investigated and clarified by means of spectroscopic approaches. The mechanism has been demonstrated to be ruled by the activation of CNPs surface chemical groups, which became active sites for the chemical reduction of the toxic ion. Further, Mn(VI) has been reduced to Mn(II), in water, without acid activation according to data reported in the literature for other carbon based-nanosystems.

## Figures and Tables

**Table 1:** Elemental composition of the coffee grounds and CNPs quantified from the XRF measurement.

Elements	Coffee Grounds		CNPs *	
	Weight %	Atomic %	Weight %	Atomic %
Ca	47.10	44.31	10.40	7.73
S	10.88	12.79	0.21	0.20
Mg	8.53	13.23	20.67	25.35
Fe	8.13	5.49	0.53	0.28
K	8.02	7.73	7.00	5.33
P	4.48	5.45	42.60	41.00
Al	3.86	5.39	17.86	19.73
Zn	3.85	2.22	0.40	0.18
Mn	2.26	1.55	0.13	0.07
Cu	2.19	1.3	0.03	0.01
Ni	0.52	0.33	0.12	0.06
Si	0.10	0.13	--	--
Ti	0.09	0.07	0.06	0.04

* Elemental contribution of silicon from the Si substrate was not considered.

## Data Availability

Data is contained within the article.

## Supplementary Materials

The following are available online at https://www.mdpi.com/article/10.3390/nano11051064/s1. Figure S1. Up-conversion fluorescence recorded at different excitation wavelengths. Figure S2. ζ-potential performed on the as-synthetized CNPs, after the interaction KMnO4, after acetic acid (1 h at pH 4.5) exposure and after acetic acid and dichromate interaction (1 h at pH 4.5). Figure S3. (a) Raman spectrum of CNPs after interaction with the chromate/dichromate solution (10^−4^ M) (b) Raman spectrum of CNPs after interaction with KMnO_4_ solution (10^−4^ M). Figure S4. FT-IR (in the 1850–800 cm^−1^ range) spectra of CNPs in presence of Mn(VII) at pH 6.6 (purple line), in presence of Cr(VI) at pH 6.6 (blue line), treated at pH 4.5 (red line), in the presence of Cr(VI) at pH 4.5 (green line). Figure S5. Down conversion fluorescence of CNPs at two different pH values and in presence of Cr(VI) and Cr(III). Figure S6. Down conversion fluorescence of CNPs and in presence of potassium permanganate and manganese(II) sulphate.
