# Supplementary material for: Coffee Grounds-Derived CNPs for Efficient Cr(VI) Water Remediation
Source: Nanomaterials (Basel). 2021 Apr 21;11(5):1064. doi: 10.3390/nano11051064 (PMC8143114; doi:10.3390/nano11051064)
Supplement: Supplementary file 1 [file nanomaterials-11-01064-s001.zip › nanomaterials-1174851-SI.pdf]

SUPPORTING INFORMATION

Coffee Grounds-Derived CNPs for Efficient Cr(VI) Water Remediation

Simona Bettini [1,2,*], Michela Ottolini [3], Rosanna Pagano [1,2], Sudipto Pal [3], Antonio Licciulli [3], Ludovico Valli [1,2] and Gabriele Giancane [2,4,*]

1. Department of Biological and Environmental Sciences and Technologies, DISTEBA, University of Salento, Via per Arnesano, I-73100 Lecce, Italy; rosanna.pagano@unisalento.it (R.P.); ludovico.valli@unisalento.it (L.V.)
2. Consorzio Interuniversitario Nazionale per la Scienza e Tecnologia dei Materiali, INSTM, Via G. Giusti, 9,
I-50121 Firenze, Italy
3. Department of Engineering for Innovation, University of Salento, Via per Monteroni, I-73100 Lecce, Italy; michela.ottolini@unisalento.it (M.O.); sudipto.pal@unisalento.it (S.P.); antonio.licciulli@unisalento.it (A.L.)
4. Department of Cultural Heritage, University of Salento, Via D. Birago, 48, I-73100 Lecce, Italy

* Correspondence: simona.bettini@unisalento.it (S.B.); gabriele.giancane@unisalento.it (G.G.); Tel.: +39-083-229-9445 (S.B.); +39-083-229-9442 (G.G.)

Citation: Bettini, S.; Ottolini, M.; Pagano, R.; Pal, S.; Licciulli, A.; Valli, L.; Giancane, G. Coffee Grounds-Derived CNPs for Efficient Cr(VI) Water Remediation. *Nanomaterials* **2021**, *11*, 1064. https://doi.org/10.3390/nano11051064

Academic Editor: Constantine D. Stalikas

Received: 23 March 2021
Accepted: 17 April 2021
Published: date

Publisher's Note: MDPI stays neutral with regard to jurisdictional claims in published maps and institutional affiliations.

Copyright: © 2021 by the authors. Submitted for possible open access publication under the terms and conditions of the Creative Commons Attribution (CC BY) license (http://creativecommons.org/licenses/by/4.0/).

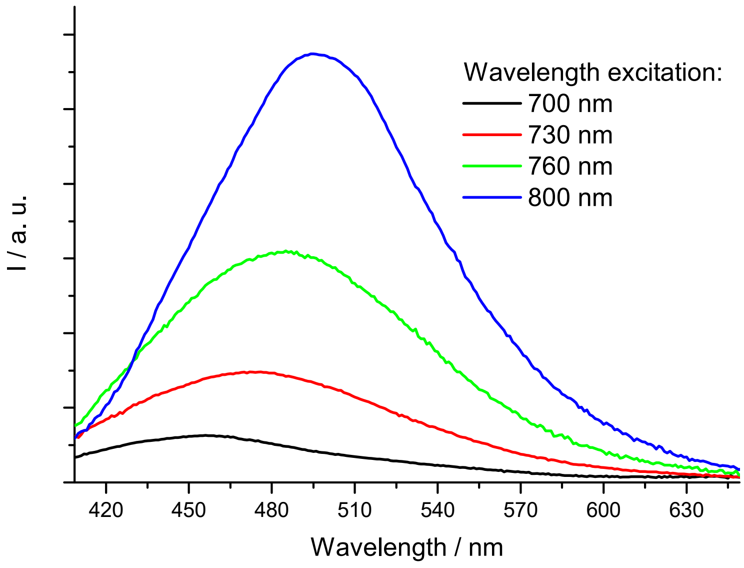

Figure S1. Up-conversion fluorescence recorded at different excitation wavelengths.

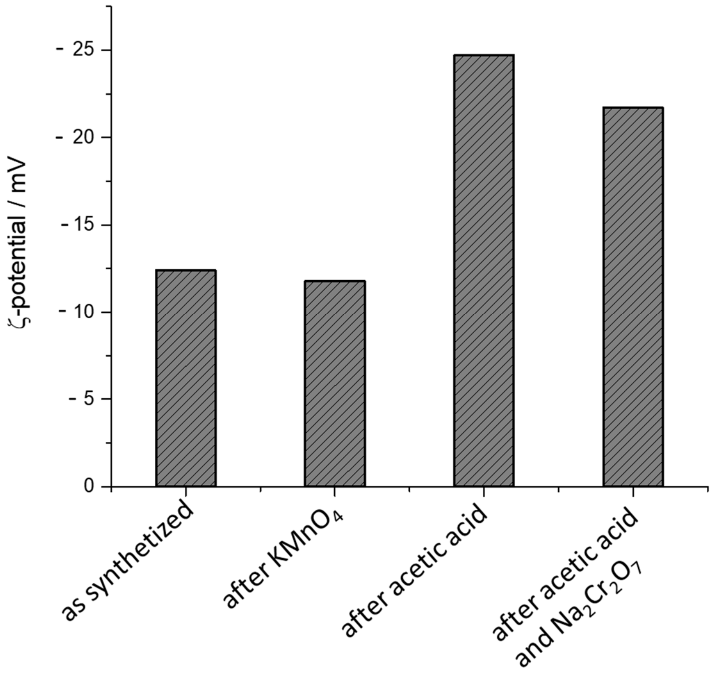

Figure S2. ζ-potential performed on the as-synthetized CNPs, after the interaction KMnO4, after acetic acid (1h at pH4.5) exposure and after acetic acid and dichromate interaction (1h at pH4.5).

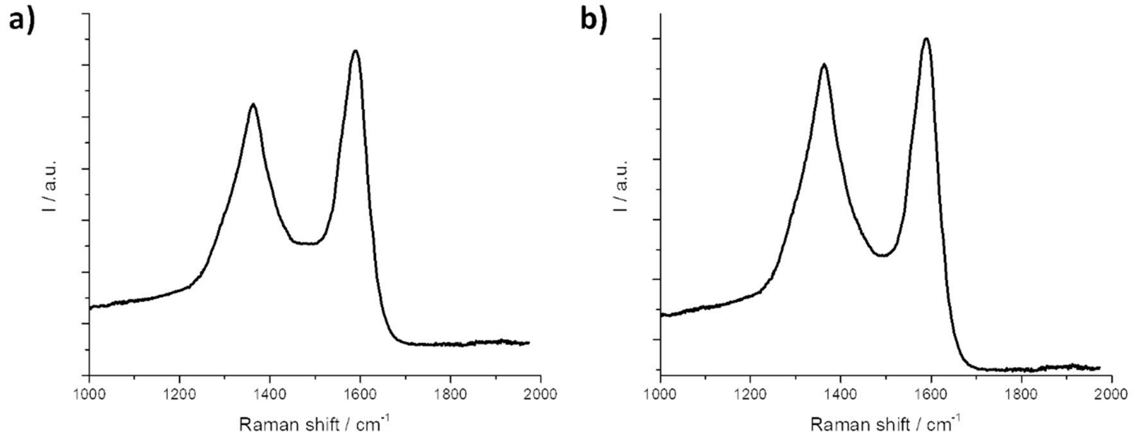

Figure S3. a) Raman spectrum of CNPs after interaction with the chromate/dichromate solution (10^{-4} M); b) Raman spectrum of CNPs after interaction with $KMnO_4$ solution (10^{-4} M).

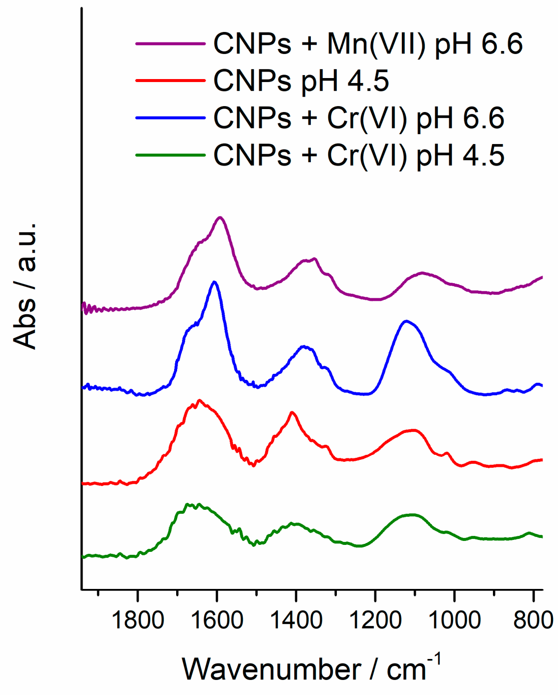

Figure S4. FT-IR (in the 1850-800 cm^{-1} range) spectra of CNPs in presence of Mn(VII) at pH 6.6 (purple line), in presence of Cr(VI) at pH 6.6 (blue line), treated at pH 4.5 (red line), in presence of Cr(VI) at pH 4.5 (green line).

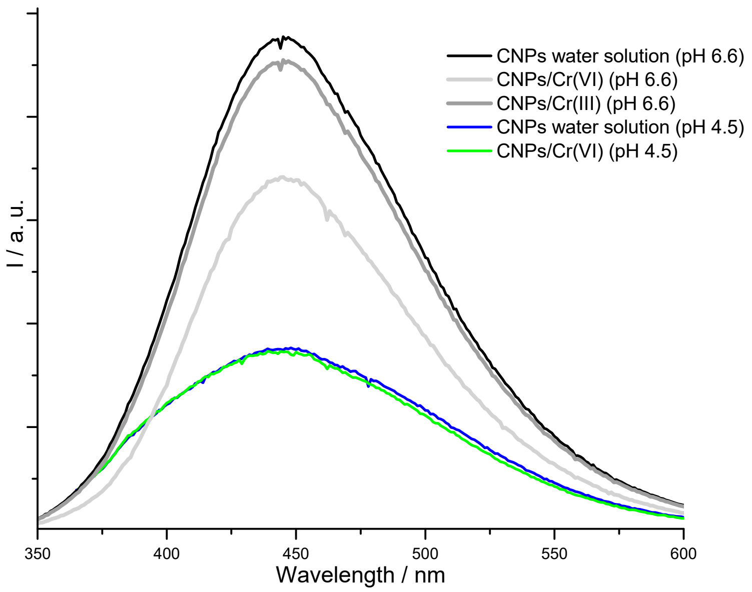

Figure S5. Down conversion fluorescence of CNPs at two different pH values and in presence of Cr(VI) and Cr(III).

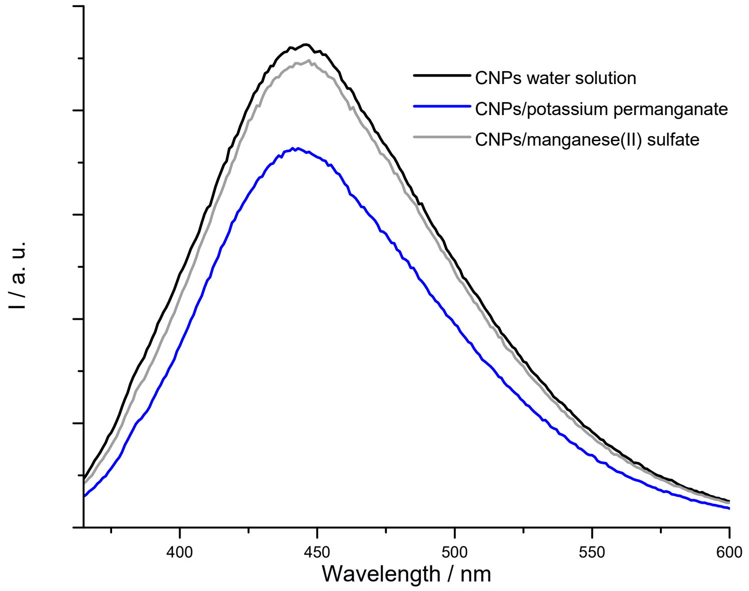

Figure S6. Down conversion fluorescence of CNPs and in presence of potassium permanganate and manganese(II) sulphate.
